# 3D star shot analysis using MAGAT gel dosimeter for integrated imaging and radiation isocenter verification of MR‐Linac system

**DOI:** 10.1002/acm2.13615

**Published:** 2022-04-18

**Authors:** Jeong Ho Kim, Bitbyeol Kim, Wook‐Geun Shin, Jaeman Son, Chang Heon Choi, Jong Min Park, Ui‐Jung Hwang, Jung‐in Kim, Seongmoon Jung

**Affiliations:** ^1^ Department of Radiation Oncology Samsung Changwon Hospital Sungkyunkwan University School of Medicine Changwon Republic of Korea; ^2^ Department of Radiation Oncology Seoul National University Hospital Seoul Republic of Korea; ^3^ Institute of Radiation Medicine Seoul National University Medical Research Center Seoul Republic of Korea; ^4^ Biomedical Research Institute Seoul National University Hospital Seoul Republic of Korea; ^5^ Department of Radiation Oncology Seoul National University College of Medicine Seoul Republic of Korea; ^6^ Robotics Research Laboratory for Extreme Environments Advanced Institute of Convergence Technology Suwon Republic of Korea; ^7^ Department of Radiation Oncology Chungnam National University Sejong Hospital Sejong Republic of Korea

**Keywords:** 3D star shot analysis, MAGAT, MR‐Linac, imaging isocenter, radiation isocenter

## Abstract

**Purpose:**

This study aims to investigate a star shot analysis using a three‐dimensional (3D) gel dosimeter for the imaging and radiation isocenter verification of a magnetic resonance linear accelerator (MR‐Linac).

**Methods:**

A mixture of methacrylic acid, gelatin, and tetrakis (hydroxymethyl) phosphonium chloride, called MAGAT gel, was fabricated. One MAGAT gel for each Linac and MR‐Linac was irradiated under six gantry angles. A 6 MV photon beam of Linac and a 6 MV flattening filter free beam of MR‐Linac were delivered to two MAGAT gels and EBT3 films. MR images were acquired by MR‐Linac with a clinical sequence (i.e., TrueFISP). The 3D star shot analysis for seven consecutive slices of the MR images with TrueFISP was performed. The 2D star shot analysis for the central plane of the gel was compared to the results from the EBT3 films. The radius of isocircle (IC_r_) and the distance between the center of the circle and the center marked on the image (IC_d_) were evaluated.

**Results:**

For MR‐Linac with MAGAT gel measurements, IC_d_ at the central plane was 0.46 mm for TrueFISP. Compared to EBT3 film measurements, the differences in IC_d_ and IC_r_ for both Linac and MR‐Linac were within 0.11 and 0.13 mm, respectively. For the 3D analysis, seven consecutive slices of TrueFISP images were analyzed and the maximum radii of isocircles (IC_r_max_) were 0.18 mm for Linac and 0.73 mm for MR‐Linac. The tilting angles of radiation axis were 0.31° for Linac and 0.10° for MR‐Linac.

**Conclusion:**

The accuracy of 3D star shot analysis using MAGAT gel was comparable to that of EBT3 film, having a capability for integrated analysis for imaging isocenter and radiation isocenter. 3D star shot analysis using MAGAT gel can provide 3D information of radiation isocenter, suggesting a quantitative extent of gantry‐tilting.

## 1 INTRODUCTION

Radiotherapy aims to kill cancer cells and shrink tumors while minimizing the radiation exposure of normal tissues. To such end, advanced techniques including intensity modulated radiation therapy (IMRT) and volumetric modulated arc therapy (VMAT), have been developed and used in clinical practice.^1^ In addition, image guided radiation therapy (IGRT) can enhance the treatment accuracy. Magnetic resonance guided radiation therapy (MRgRT) based on magnetic resonance imaging (MRI) has been increasingly favored due to the advantage of high soft tissue contrast compared to the conventional IGRT based on X‐ray.^2^, ^3^ The advancement of techniques has further complicated the treatment procedures with a potential increase in errors. Thus, the quality assurance (QA) of radiation therapy machine and imaging device is important. The American Association of Physicists in Medicine (AAPM) provides a QA guideline through task group 142 (TG‐142).

Isocenter accuracy is of central importance in enhancing the treatment accuracy of any given radiotherapy machine. In general, for linear accelerator (Linac), the isocenter accuracy has been verified by Winston‐Lutz test or star shot analysis using radiochromic films. The film‐based star shot tests use several beams that are irradiated from various angles of gantry, collimator, and couch.^4^ This method has proven to be highly effective in determining the coincidence of the mechanical isocenter with room laser indication and the radiation isocenter with film exposure. In the case of MR‐Linac or MR‐cobalt for conducting MRgRT, the final patient set‐up involves acquisition of MR images. Thus, the coincidence of the imaging isocenter and radiation isocenter is important. In 2019, a method to verify the imaging and radiation isocenter using an ionization chamber array was presented.^5^ Elekta Unity (Elekta AB, Stockholm, Sweden) system uses ZrO_2_ spherical phantom and electronic portal imaging device (EPID) to check the coincidence of imaging isocenter and radiation isocenter.^6^ However, EPID is not applicable at the other MR‐Linac system such as MRIdian (ViewRay, Inc., OH, USA). Usually, a radiochromic film is used to determine the isocentricity and to confirm the coincidence of the imaging isocenter and the radiation isocenter as well as the coincidence of the virtual isocenter and the radiation isocenter. Subsequently, a phantom that can be analyzed by MRI is used to confirm that the imaging isocenter is coincident with the virtual isocenter.^7^ Kim et al. reported image registration‐based quantification for MRI isocenter verification in clinical RT mode using ViewRay cylindrical phantom and radiochromic film.^8^

However, only a 2D isocenter size and location can be obtained from a 2D star shot measurement, while the isocenter size and location have 3D information. In other words, a 2D star shot did not allow for isocenter alignment measurements in 3D.^9^, ^10^ 3D information about the isocenter size and location can be obtained from several 2D star shots under the assumption that the uncertainty of the repeated positioning of films is almost negligible.^11^ These film measurements is very labor intensive and time consuming.^11^ Recently, a three‐dimensional (3D) analysis for evaluating isocentricity has been suggested to overcome the limitation of the 2D analysis by film measurements. Velten et al. proposed a 3D QA tool using PRESAGE^®^ and verified the plausibility of PRESAGE^®^.^11^ They provide a more comprehensive view on the isocenters of Linac than the 2D film method, suggesting a 3D position and size of isocenter.^11^ However, typical analysis with PRESAGE^®^ uses optical CT to read dose profiles so that PRESAGE^®^ dosimeter is not suitable for analyzing the accuracy of radiation isocenter for MR‐Linac. Tsuneda et al. studied 3D QA tool based on the scintillator imaging system for verification of 3D isocentricity and direct evaluation of the sagging angle using plastic scintillator and CCD camera.^12^

More recently, Dorsch et al. proposed a 3D QA tool for the isocenter verification of MR‐Linac using the polyacrylamide gel and THPC (PAGAT) gel, and they reported that the developed phantom with PAGAT gel could analyze the isocentricity of the irradiation, the alignment of the irradiation and imaging isocenter, and 3D MR image distortion in a single measurement.^9^, ^10^ Polymer gels upon radiation incidence allow reactions among monomers that generate free radicals and form polymers. Formation of polymers can be visualized using various imaging modalities such as ultrasound, optical computed tomography, X‐ray computed tomography (CT), and MRI.^13^, ^14^, ^15^, ^16^, ^17^ Furthermore, several studies have reported that polymer gels can be potentially used for 3D dosimetry especially for MRgRT.^18^, ^19^, ^20^, ^21^

In the previous study reported by Dorsch et al.,^10^ an imaging sequence for the star shot analysis was a T2‐weighted turbo spin echo (T2w‐TSE) research sequence, which limited the application by general users in the MR‐Linac system. It also takes long operation time for image acquisition (approximately 40 min to 105 min). In addition, it is reported that an acrylamide in PAGAT gel has severe toxicity; LD_50_ for mice is known as 0.17 g/kg.^22^

In this study, to overcome the aforementioned limitations of the conventional QA tool and previous studies using PAGAT measurement, a 3D QA tool using MAGAT (methacrylic acid gelatin gel and THPC) gel and MR‐Linac with a fast imaging sequence was developed for measurement of isocentricity of the radiation. MAGAT gel (a mixture of MAA, gelatin, and tetrakis (hydroxymethyl) phosphonium chloride [THPC]) exhibits less toxicity than that of polymer gel using acrylamide or acrylic acid as monomers (i.e., PAGAT gel). LD_50_ for mice of methacrylic acid (MAA) in MAGAT gel is 8.4 g/kg and it is less toxic than acrylamide in PAGAT gel.^22^ Another advantage is the higher radiation sensitivity than that of other polymer gels such as MAGAS, MAGIC, PAGAT, VIPAR, HEA.^22^, ^23^, ^24^ Additionally, the MAGAT gel can be manufactured in‐house at a low cost under conditions of atmospheric pressure, temperature, and light. In addition, this study also aims to evaluate a utility of MAGAT gel for star shot analysis for conventional Linac to assess radiation isocentricity. 3D information obtained from a star shot analysis using MAGAT gel for Linac can provide a titling angle of gantry of Linac as well as the size and location of isocenter in 3D.

## 2 MATERIALS AND METHODS

### 2.1 Fabrication of MAGAT gel phantom

The MAGAT gel was fabricated at normal atmospheric condition. It should be noted that oxygen contamination often occurs during gel preparation under atmospheric conditions. The MAGAT gels without oxygen contamination were used for the analysis. The MAGAT gel was composed of 5% w/w gelatin, 6% w/w MAA, 10 mM THPC, and 89% w/w deionized water, as reported by Razak et al. to provide the highest sensitivity.^24^ Gelatin was slowly poured into the deionized water heated to 80°C on a hot plate and magnetic stirrer device, and the mixture was continuously stirred using a magnetic bar until complete dissolution to produce a transparent solution. The temperature of gelatin solution must be kept as close as room temperature (i.e., 25°C) when mixing the monomers to avoid thermal‐polymerization that may be caused due to the temperature of the solution.^25^ The gelatin solution was stirred after cooling, at which MAA and THPC were added and stirred. The gel solution was placed in the customized acrylic phantom and stored in a 4°C refrigerator for 24 h prior to an irradiation. The gel was left at room temperature for 30 min before irradiation.^26^

**FIGURE 1:**
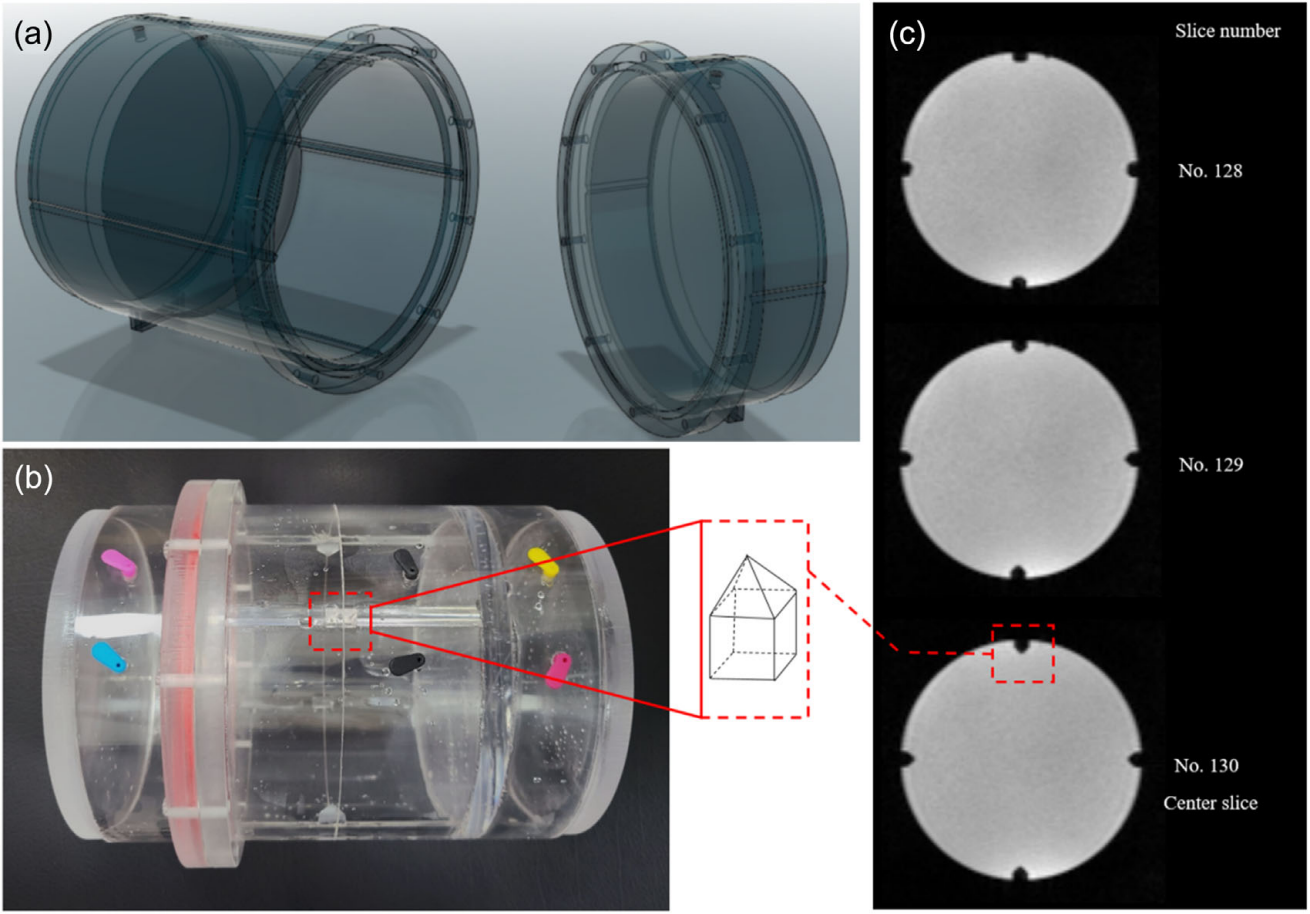
(a) Structural design and (b) external form of the acrylic phantom for 3D MAGAT gel, and (c) MR image taken after the filling of MAGAT gel

Figure 1 shows the basic structure and external form of the acrylic phantom fabricated in this study as well as the MR image. The phantom had a length of 19.5 cm in length and diameter of 15 cm. The MAGAT gel had a length of 8.5 cm and diameter of 14 cm. Two cylinders with 5 cm height at either side of the MAGAT gel were filled with water. To adjust the center of the QA phantom to the laser‐marked virtual isocenter, the geometrical center of the phantom was marked on the surface with a line, and inside the phantom, four tetrahedron markers were placed for the verification of the virtual isocenter and imaging isocenter on the MR image. As shown in Figure 1c, the tetrahedron markers in the center slice were the largest among the image slices, and the size of the marker decreased as the distance from the center slice increased.

### 2.2 Irradiation

**FIGURE 2:**
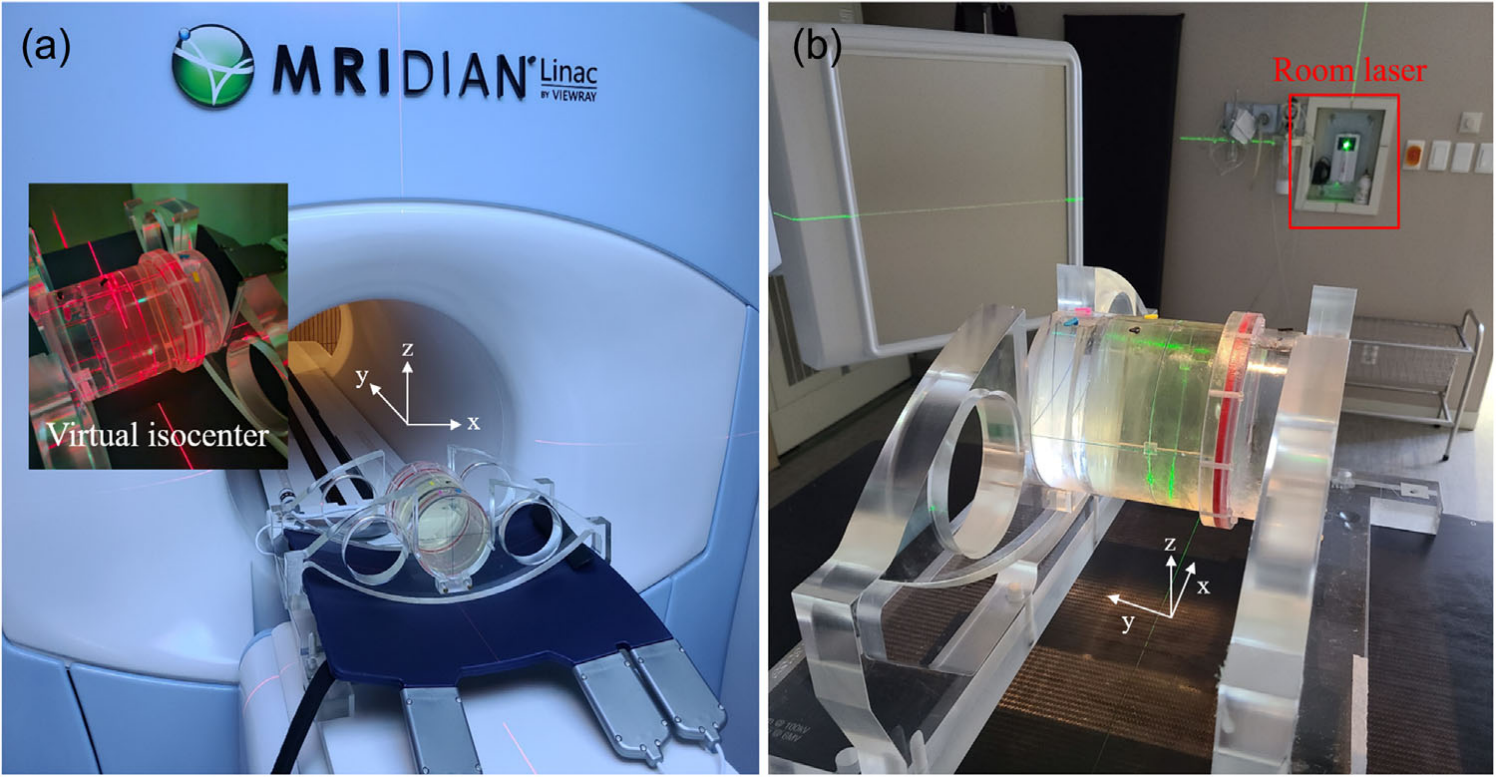
Experimental setup of the isocenter measurement based on MAGAT gel phantom for (a) MR‐Linac and (b) Linac

Figure 2 shows the experimental set‐up based on the MAGAT gel phantom. The measurement of isocenter was conducted for the MR‐Linac system (0.35T MRIdian, ViewRay, Inc., OH, USA), and the Linac machine (VitalBeam, Varian Medical Systems, Palo Alto, CA). The experimental setup for the MR‐Linac is shown in Figure 2a. The center of the phantom was aligned to the virtual isocenter indicated by the room laser. Then the migration of 155 cm along the *y*‐axis to the treatment isocenter was conducted. The irradiation energy was a 6 MV flatting filter free photon beam, with a source‐to‐axis distance (SAD) of 90 cm and dose rate of 600 MU min^–1^. The radiation beam was from six angles, 29°, 110°, 160°, 190°, 230°, and 315°, and 500 MU was irradiated with a 0.5 × 24 cm^2^ field size at each angle. The experimental setup for the Linac is shown in Figure 2b. The central line marked on the exterior of the MAGAT gel phantom was aligned to the mechanical isocenter of the machine indicated by the room laser. The irradiation energy was a 6 MV photon beam, with 100 cm SAD, 600 MU min^–1^ dose rate, and 0.5 × 24 cm^2^ field size. The radiation beam was from six angles, 30°, 110°, 160°, 190°, 230°, and 315°, and 500 MU was irradiated at each angle. Instead of 30°, 29° was selected for MR‐Linac, since the angles between 30° and 33° are not available due to technical limitations.^27^ The irradiation of the Gafchromic external beam therapy (EBT3) film was performed in identical conditions, and the film was placed in a ViewRay Daily QA phantom (ViewRay Inc. Cleveland, OH, USA.)

### 2.3 Imaging

After the star shot irradiation in the Linac, the MAGAT gel phantom was set to the virtual isocenter in the MR‐Linac for MR image scanning. The MAGAT gel for measurement of isocenter of Linac might have a potential repositioning uncertainty due to the temporal and spatial difference between the irradiation position and readout position. To reduce the potential repositioning error, we checked the room lasers carefully for both irradiation and imaging procedures. We assumed that the repositioning error was negligible in this study. The MAGAT gel phantom after the star shot irradiation in the MR‐Linac was scanned using the same device without any change in the setup. Signal of polymer gels continues to increase up to 24 h after the irradiation, improving a contrast‐to‐noise ratio (i.e., high dose response).^10^, ^26^ However, we investigate only geometrical information so that a conversion into dose is not required. In the previous study by Dorsch et al.,^10^ the PAGAT gel was imaged directly after the irradiation. In this study, MR scans were performed 30 min after the irradiation and we can observe sufficient polymerization to investigate geometrical information. MR scans were performed with two different sequences. A true fast imaging sequence (denoted as TrueFISP) was used with a steady state precession sequence (bSSFP), yielding a T2/T1‐weighted contrast. The resolution of TrueFISP images was 1.5 × 1.5 × 1.5 mm^3^, with an imaging time of 2 min 8 s and field of view (FOV) of 400 × 430 × 400 mm^3^. The other sequence using T2‐weighted contrast (denoted as T2‐w) had a resolution of 1 × 1 × 2.5 mm^3^, imaging time of 76 min 50 s, and FOV of 250 × 250 × 27.5 mm^3^. The EBT3 films were scanned 30 min after the irradiation by using an Epson 10000XL flatbed scanner with transmission mode resolution of 0.08 × 0.08 mm^2^.

### 2.4 Star shot analysis

The in‐house code for a star shot analysis was developed using MATLAB R2020b. The methodologies for the analysis have been reported in other publications.^28^, ^29^ To exclude the superposition area of the beam in the analysis, eight concentric circumferences were produced from a point 4.2 cm away from the center up to 5.25 cm in terms of radial distance. Two valleys (one for entrance beam, and the other for exit beam) of each irradiation angle were obtained for each circumference, resulting in 16 valleys per angle. A single profile consisted of 12 valleys (two valleys per angle × six angles per one circumference) per circumference. Pixel positions of eight profiles were expressed in a unit of degree, as the pixel values were interpolated using bilinear interpolation with a step size of 0.18°. Subsequently, the central position of beam was defined as the center of full width half‐maximum (FWHM) of each valley. The central line per beam angle was then obtained by fitting a straight line along the points of the eight central points in the entrance beam and eight central points in the exit beam. The inner circles of all crossing central lines were estimated, and the smallest intersecting circle was selected by using the analytic solution suggested by Depuydt et al.^29^ The radius of this circle was denoted as IC_r_, and the distance between the center of the circle and the center marked on the image was IC_d_. Particularly, IC_d_ for the Linac star shot analysis indicates the distance between the radiation isocenter and the mechanical isocenter, while IC_d_ for the MR‐Linac indicates the distance between the radiation isocenter and the imaging isocenter. IC_d_ and IC_r_ for EBT3 film and for the central MR image slice with T2‐w were analyzed, while IC_d_ and IC_r_ for seven consecutive MR image slices with TrueFISP sequence were analyzed for the measurement of isocenter accuracy for MR‐Linac. Furthermore, lateral profiles of each entrance beam were evaluated as the average over eight concentric circumferences located at a radial distance of 4.2–5.25 cm from the isocenter. The minimum position of the entrance beam was determined as the average of the minimum positions of the individual circumferences.

**FIGURE 3:**
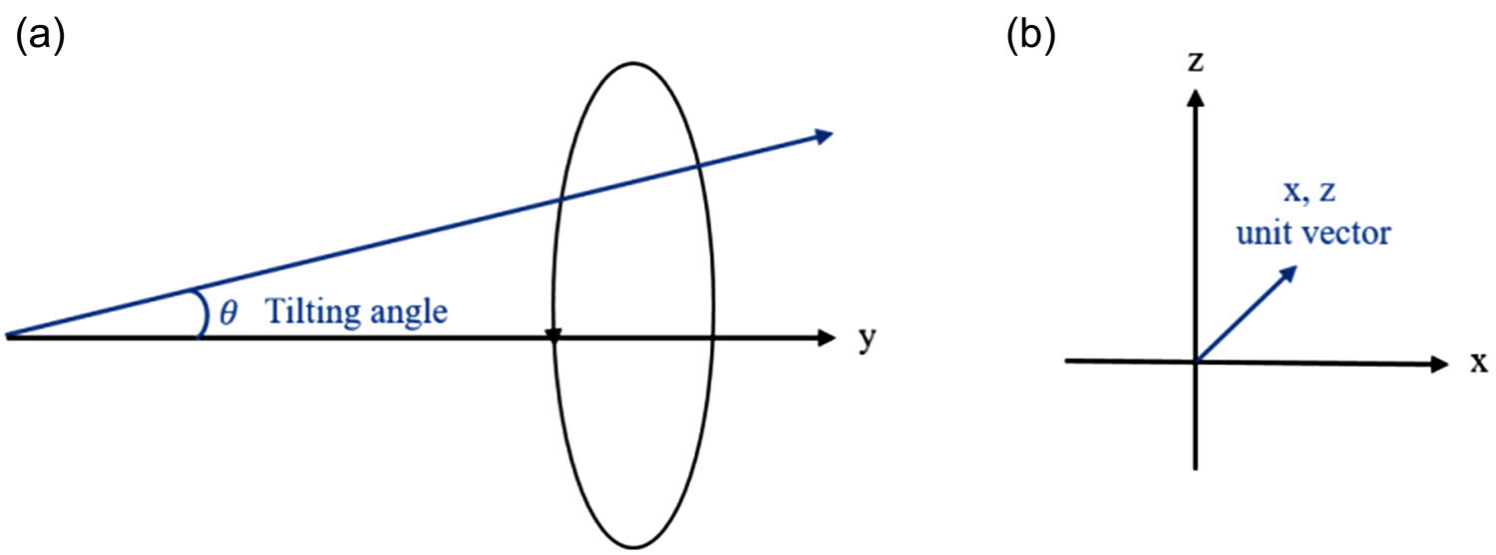
(a) The gantry‐tilting angle between the y‐direction (see Figure 2) and the radiation axis of gantry rotation and (b) *x*‐ and *z*‐directional unit vector of radiation axis of gantry rotation

For the gantry‐tilting analysis using the 3D star shot measurement, we generated a single linear regression curve in 3D space by fitting the isocenters from seven consecutive image slices. Figure 3a indicates tilting angle between the y‐direction (Figure 2) and radiation axis of gantry rotation. Figure 3b describes *x*‐ and *z*‐directional unit vector of the radiation axis of gantry rotation.

## 3 RESULTS

**FIGURE 4:**
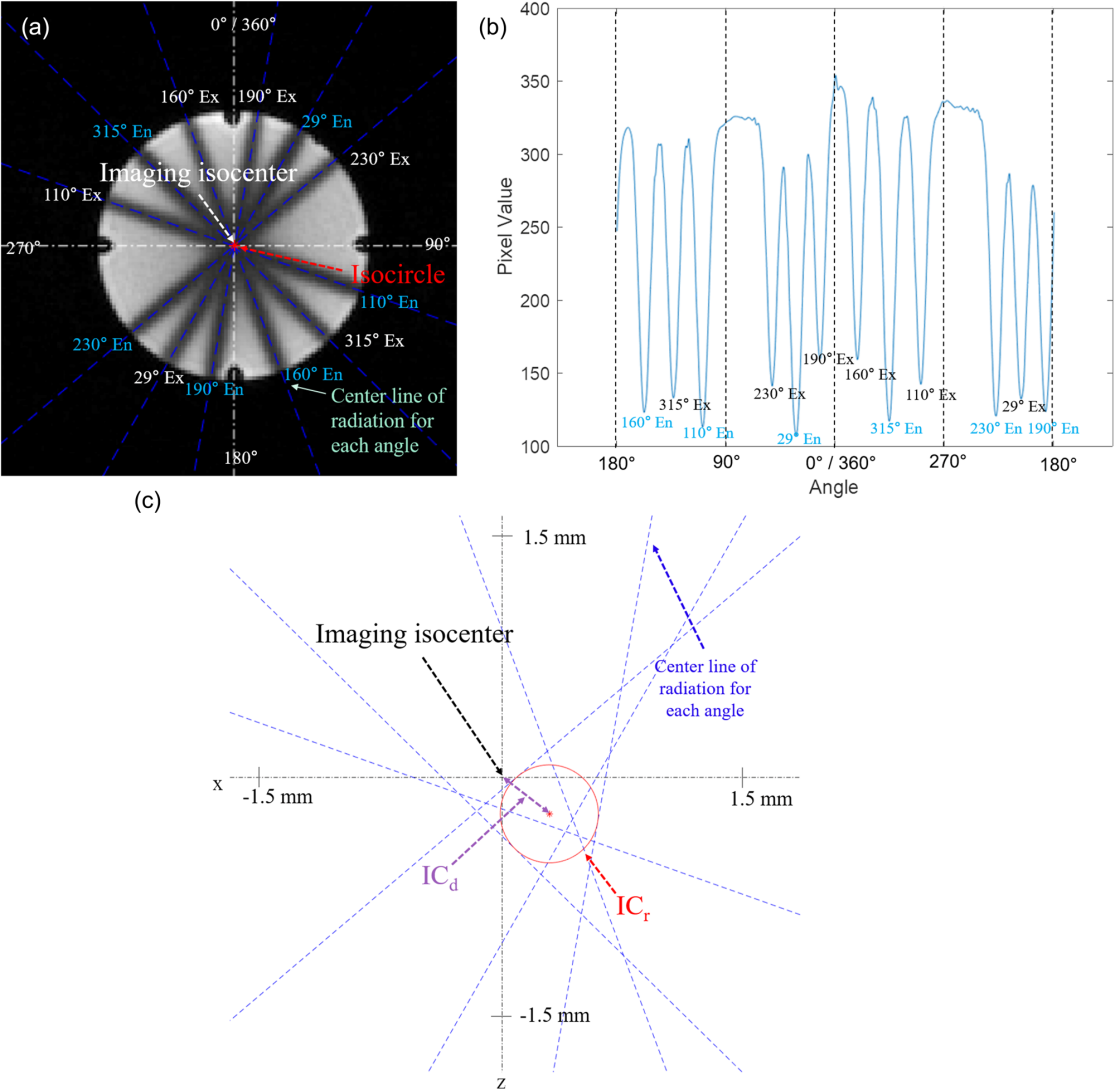
(a) MR image of MAGAT gel scanned with TrueFISP, (b) a profile of entrance beams (denoted as En) and exit beams (denoted as Ex), and (c) IC_d_ and IC_r_ for Linac

Figure 4 shows the TrueFISP image of the MAGAT gel phantom obtained after the star shot irradiation in Linac, illustrating the central lines of entrance beams (denoted as En in Figure 4) and exit beams (denoted as Ex in Figure 4). The profile of the 8^th^ circumference consisting of twelve valleys (six for entrance beams and six for exit beams) was shown in Figure 4b. Figure 4c illustrates the imaging isocenter, the central lines of the entrance beams and the exit beams, IC_d_, and IC_r_. The white dashed line in Figure 4a and the black dashed line in Figure 4c indicate the imaging isocenter, whereas the blue dashed line indicates the central line of each beam. The red circle indicates the minimum tangential circle inscribed in the central line of each beam.

**FIGURE 5:**
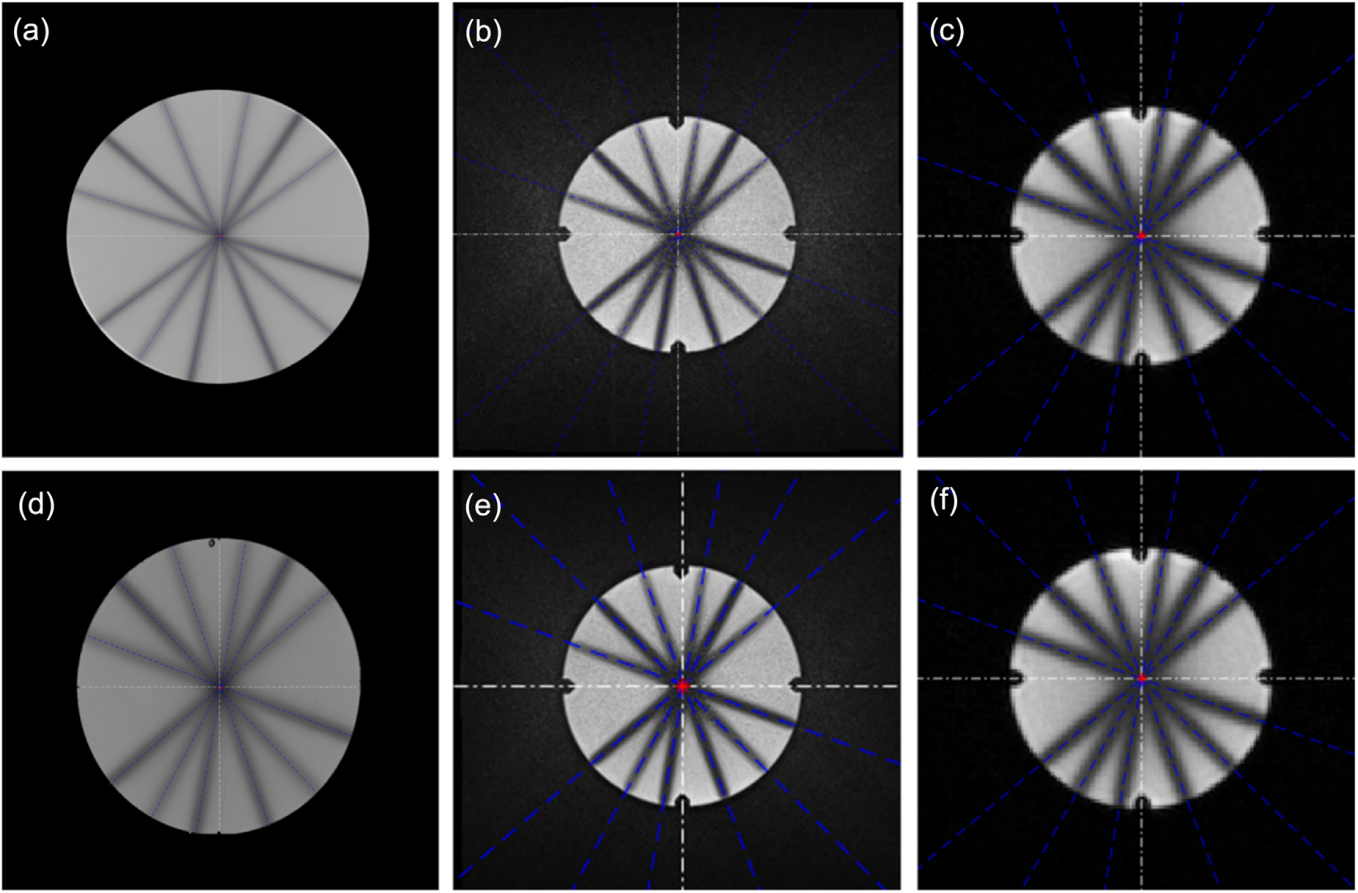
(a) Scanned image of EBT3 film, (b) MR image with T2‐w, and (c) MR image with TrueFISP of MAGAT gel for Linac; (d) scanned image of EBT3 film, (e) MR image with T2‐w, and (f) MR image with TrueFISP sequence of MAGAT gel for MR‐Linac. The blue dashed line is central line for each angle. The white dashed line is imaging isocenter and the red circle is isocircle

Figure 5 shows the scanned images of MAGAT gel and EBT3 film after the star shot irradiation in the Linac and MR‐Linac. Table 1 presents the results of star shot analysis for Linac and MR‐Linac, where *x* and *z* are the coordinates of the radiation isocenter. For central slice results of Linac, the IC_d_ was 0.07 mm for the EBT3 film. In addition, Table 1 also presents the results of the star shot analysis of seven consecutive slices of MAGAT gel with TrueFISP sequence for the isocenter measurement in Linac and MR‐Linac.

**TABLE 1:** Star shot analysis results for Linac and MR‐Linac

Linac					
QA tool	Slice	*x* (mm)	*z* (mm)	IC_d_ (mm)	IC_r_ (mm)
EBT3 Film	central slice	0.07	−0.01	0.07	0.11
MAGAT with T2‐w	4 (central slice)	0.07	−0.14	0.16	0.15
MAGAT with TrueFISP	1	0.09	−0.06	0.11	0.13
	2	0.09	−0.07	0.12	0.13
	3	0.10	−0.06	0.12	0.15
	4 (center slice)	0.10	−0.07	0.12	0.15
	5	0.10	−0.09	0.13	0.13
	6	0.09	−0.09	0.13	0.11
	7	0.11	−0.11	0.15	0.12
			Mean (mm)	0.13 ± 0.01	0.13 ± 0.01

MR‐Linac					
	Slice	*x* (mm)	*z* (mm)	IC_d_ (mm)	IC_r_ (mm)
EBT3 Film	central slice	0.43	0.33	0.54	0.57
MAGAT with T2‐w	4 (central slice)	0.39	0.18	0.43	0.53
MAGAT with TrueFISP	1	0.46	0.16	0.49	0.67
	2	0.41	0.19	0.45	0.67
	3	0.41	0.18	0.45	0.67
	4 (center slice)	0.42	0.20	0.46	0.70
	5	0.43	0.20	0.48	0.68
	6	0.42	0.20	0.47	0.63
	7	0.45	0.17	0.48	0.58
			Mean (mm)	0.47 ± 0.01	0.65 ± 0.01

**FIGURE 6:**
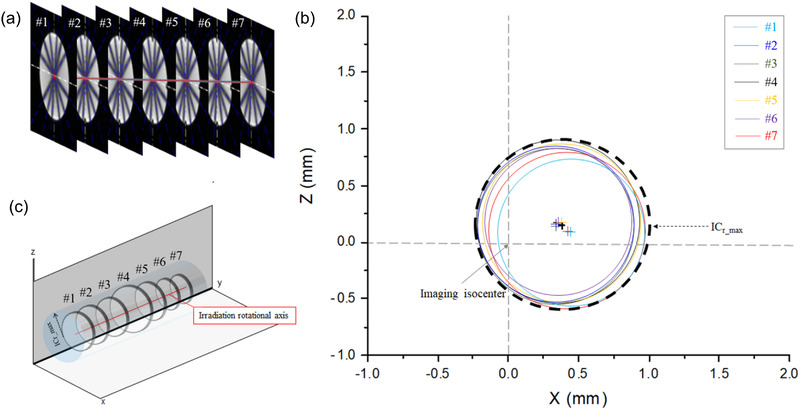
(a) A stack of MR images with TrueFISP sequence of MAGAT gel, (b) isocenter positions and isocircles presented in a single *x*–*z* plane, and (c) 3D view of isocircles with radiation axis for MR‐Linac

**FIGURE 7:**
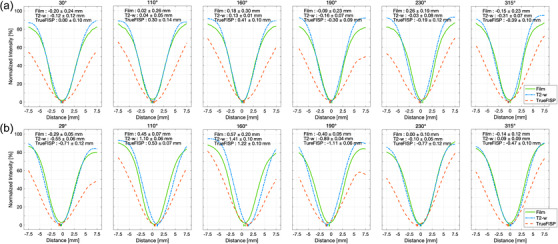
The averaged lateral profiles measured by EBT3 film, MAGAT gel with T2‐w, and MAGAT gel with TrueFISP for (a) Linac and (b) MR‐Linac. The mean with standard deviation (1σ) of the minimum positions is written in the figures and as error bars for EBT3 film, MAGAT gel with T2‐w, and MAGAT gel with TrueFISP

Figure 6 shows the 3D star shot analysis results from the MR‐Linac measurement. The IC_d_ and IC_r_ of seven slices in single *xz*‐plane representation are shown in Figure 6b, with the radius of isocylinder (IC_r_max_) including the isocircles of all slices. The 3D representation of Figure 6b is shown in Figure 6c, which can be a cylinder with IC_r_max_ of 0.18 mm in Linac and 0.73 mm in MR‐Linac. Figure 7 illustrates the average lateral profiles of eight circumferences for the six beam angles with Linac and with MR‐Linac. The minimum positions for the six beam angles for EBT3, T2‐w, and TrueFISP were also shown in the figures. The pixel values of EBT3, T2‐w MR image, and TrueFISP image were rescaled to 0–100 (%). The profiles from EBT3 and MAGAT gel resulted in comparable minimum positions. The differences may be due to the alignment uncertainties.

**FIGURE 8:**
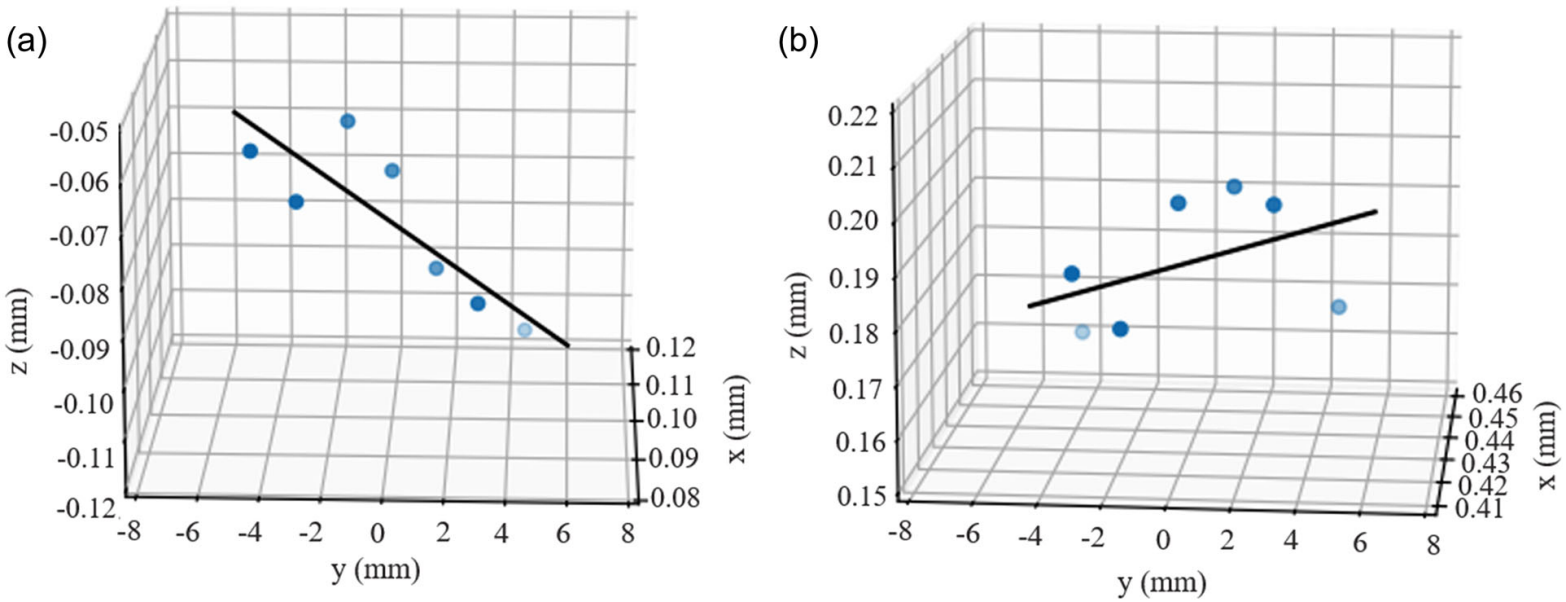
Linear regression curves of 3D star shot results for (a) Linac and (b) MR‐Linac

Figure 8 shows linear regression curves indicating the radiation axis due to the combined effect of gantry tilting. The angles of tilting were 0.31° and 0.10° with *y*‐direction (longitudinal direction) in Linac and MR‐Linac, respectively. The *x*‐, *z*‐directional unit vector of tilting in *xz*‐plane is (−0.26, 0.97) and (−0.14, −0.99) in Linac and MR‐Linac, respectively. The R^2^ of the generated curves for Linac and MR‐Linac was greater than 0.99.

## 4 DISCUSSION

In this study, the 3D star shot analysis for the isocenter verification for Linac and MR‐Linac using the MAGAT gel was investigated. All IC_d_ and IC_r_ did not exceed tolerance limits of 1 mm (SRS/SBRT machine) and 2 mm (IMRT machine), as recommended by the TG‐142. For central slice results of Linac, compared to EBT3 film measurement, the IC_d_ differences were 0.09 mm for T2‐w and 0.05 mm for TrueFISP. The difference of IC_r_ between EBT3 and MAGAT was 0.04 mm for both T2‐w and TrueFISP images. The mean IC_d_ and IC_r_ were both 0.13 ± 0.01 mm. For central slice results of MR‐Linac, compared to EBT3 film measurement, the IC_d_ differences were 0.11 mm for T2‐w and 0.08 mm for TrueFISP. The differences of IC_r_ of MAGAT compared to EBT3 film images were 0.04 mm for T2‐w and 0.13 mm for TrueFISP. The mean IC_d_ of seven slices was 0.47 ± 0.01 mm. The IC_r_ at the central slice was 0.70 mm, and the mean IC_r_ was 0.65 ± 0.04 mm.

The 3D star shot analysis using MAGAT gel leads to highly effective use based on a simple measurement procedure compared to film measurement, although the fabrication of MAGAT gel might need complex procedures. Two separate steps are required when 2D film measurement is used. One should verify the coincidence of radiation isocenter and virtual isocenter using 2D film measurements. Then, it is required to verify the coincidence of imaging and the virtual isocenter using a phantom with an internal landmark that is visible on the MR image, such as the ViewRay Daily QA phantom. In contrast, the developed MAGAT gel phantom can reduce the set‐up error and measurement time as it has both the landmark for MR imaging and the radiosensitive 3D gel within a single QA tool. Thus, one setup allows the integrated measurement of the isocentricity and coincidence of imaging isocenter, and radiation isocenters.

In clinical practices, the mechanical inaccuracies and non‐absolute rigidity of the rotational component cause the rotational axis to take a 3D form of intersection rather than a single point.^11^ 2D information at each slice was used to determine the maximum and mean IC_r_ for the isocircles along the *y*‐axis, as shown in Figures 5c and 6, describing 3D information of isocenters. The previous study by Dorsch et al.^10^ suggested detecting a potential inclination of the beam due to gantry‐tilting for each beam angle using trigonometric relationship at the MRIdian system. The difference of the y‐positions between the entry beam and the exit beam was used to derive the gantry‐tilting angle at each beam angle. Contrast to the previous study by Dorsch et al.,^10^ we drew a single line with a linear regression method using the isocenters at each slice to evaluate the combined gantry tiling considering the gantry‐tilting at each beam angle. The combined gantry‐tilting angle for MR‐Linac in our study was 0.10°, while the inclinations detected by Dorsch et al.^10^ were from −0.37° to 0.19°. We evaluated not only gantry‐tilting angle but also direction vector of gantry‐tilting for Linac and MR‐Linac. Therefore, our results could provide more comprehensive information of gantry tilting. A 3D position of the isocenter can be assessed if an irradiation field size in *y*‐axis is reduced to analyze the dose profile as reported by other publications.^9^, ^10^, ^11^

In the presence of a magnetic field, IC_r_ of MR‐Linac system can be greater than the conventional Linac because the Lorentz‐force systematically deflects the secondary electrons to the same direction with respect to the beam axis.^9^ It has been reported that IC_r_ for PAGAT gel was increased by up to 0.97 mm with 1.0 T magnetic fields compared to zero magnetic fields.^9^ Magnetic fields also affects ICr of EBT3 film measurements.^30^ IC_r_ for EBT3 film was changed from 0.39 mm with zero magnetic fields to 1.37 mm with 1.0 T magnetic fields.^9^ In this study, although we did not directly compare an effect of Lorentz‐force on IC_r_ between zero magnetic field and 0.35 T magnetic field, we could at least find that the mean IC_r_ (0.65 mm) in MR‐Linac was similar to the result of the previous publication^10^ and was greater than that of the Linac (0.13 mm). For MR‐Linac, IC_d_ for MAGAT gel was smaller than that of EBT3 film. This difference might be caused by difficulties in aligning the MAGAT gel phantom and EBT film phantom accurately to the virtual isocenter, since IC_d_ is rarely affected by the presence of the magnetic field.^9^

## 5 CONCLUSION

In this study, a 3D star shot QA was investigated for an integrated isocenter verification of MR‐Linac using the MAGAT gel. The results were compared to the results from conventional 2D film measurement and showed comparable results for the central 2D MR image. 3D MAGAT gel was acquired by both the rapid scan time with the TrueFISP sequence of spatial resolution of 1.5 mm and a research sequence of superior spatial resolution of 1 mm. Isocenter accuracy was analyzed to be 0.65 ± 0.04 for 7 consecutive slices, having a combined gantry‐tilting of 0.10°. This study also reported the utility of MAGAT gel for 3D star shot analysis for Linac. Isocenter accuracy was found to be 0.13 ± 0.01 for 7 consecutive slices, having the combined gantry‐tilting of 0.10°.

## CONFLICT OF INTEREST

The authors have no relevant conflict of interest to disclose.

## AUTHOR CONTRIBUTIONS

Jung‐in Kim and Seongmoon Jung contributed equally to this work.
